# Enhanced anti-mammary gland cancer activities of tamoxifen-loaded erythropoietin-coated drug delivery system

**DOI:** 10.1371/journal.pone.0219285

**Published:** 2019-07-10

**Authors:** Chaw Yee Beh, Abdullah Rasedee, Gayathri Thevi Selvarajah, Latifah Saiful Yazan, Abdul Rahman Omar, Jia Ning Foong, Chee Wun How, Jhi Biau Foo

**Affiliations:** 1 Institute of Bioscience, Universiti Putra Malaysia, UPM Serdang, Selangor, Malaysia; 2 Faculty of Veterinary Medicine, Universiti Putra Malaysia, UPM Serdang, Selangor, Malaysia; 3 Faculty of Medicine, Universiti Putra Malaysia, UPM Serdang, Selangor, Malaysia; 4 Centre for Pre-University Studies, Faculty of Pharmacy, MAHSA University, Jenjarom, Kuala Langat, Selangor, Malaysia; 5 School of Pharmacy, Monash University Malaysia, Bandar Sunway, Selangor, Malaysia; 6 School of Pharmacy, Faculty of Health and Medical Science, Taylor’s University, Subang Jaya, Selangor, Malaysia; Columbia University, UNITED STATES

## Abstract

Nanomedicine is an emerging area in the medical field, particularly in the treatment of cancers. Nanostructured lipid carrier (NLC) was shown to be a good nanoparticulated carrier for the delivery of tamoxifen (TAM). In this study, the tamoxifen-loaded erythropoietin-coated nanostructured lipid carriers (EPO-TAMNLC) were developed to enhance the anti-cancer properties and targetability of TAM, using EPO as the homing ligand for EPO receptors (EpoRs) on breast cancer tissue cells. Tamoxifen-loaded NLC (TAMNLC) was used for comparison. The LA7 cells and LA7 cell-induced rat mammary gland tumor were used as models in the study. Immunocytochemistry staining showed that LA7 cells express estrogen receptors (ERs) and EpoRs. EPO-TAMNLC and TAMNLC significantly (p<0.05) inhibited proliferation of LA7 in dose- and time-dependent manner. EPO-TAMNLC induced apoptosis and G_0_/G_1_ cell cycle arrest of LA7 cells. Both drug delivery systems showed anti-mammary gland tumor properties. At an intravenous dose of 5 mg kg^-1^ body weight, EPO-TAMNLC and TAMNLC were not toxic to rats, suggesting that both are safe therapeutic compounds. In conclusion, EPO-TAMNLC is not only a unique drug delivery system because of the dual drug-loading feature, but also potentially highly specific in the targeting of breast cancer tissues positive for ERs and EpoRs. The incorporation of TAM into NLC with and without EPO coat had significantly (p<0.05) improved specificity and safety of the drug carriers in the treatment of mammary gland tumors.

## Introduction

Although current strategies most often used to combat breast cancers include radiation, hormonal, and targeted therapy, adjuvant chemotherapies are always incorporated to increase the rate of patient survival [1]. The most commonly used drugs in the treatment of breast cancers, doxorubicin, tamoxifen citrate, paclitaxel, and etoposide, are fraught with lack of specificity and induction of drug resistance [2]. The development of resistance to these drugs may be the result of cancer cell heterogeneity, DNA damage repair mechanism, drug efflux, and cell death inhibition [3]. In addition, most anticancer drugs are administered orally and their therapeutic concentrations in blood are governed by the efficiency of gastrointestinal tract absorption. Oral drugs administrations often need high doses to achieve therapeutic concentrations in blood.

Many chemotherapeutics are poorly water-soluble, making it difficult to achieve the desired systemic drug concentration for therapeutic efficaciousness [4]. These drugs are often not selective for cancers, instead they affect all highly proliferative cells, include those of the skin and bone marrow [5, 6]. Low water-solubility and lack of selectivity of drugs are now being addressed via several means, including conversion to prodrugs, complexation of drugs with soluble carriers, and the use of surfactants and co-solvents in the formulations [7]. Drugs in carriers administered parenterally, especially via venous route, would exhibit more predictable pharmacokinetics while reducing the toxicity often associated with the use of free drugs and gastrointestinal applications [7].

Nanomedicine has provided amicable solutions to the solubilization of drugs through the use of biologically compatible lipid nanoparticulated drug carriers. Among these lipid nanoparticles are the nanostructured lipid carriers (NLCs), formulated with solid and liquid lipids to form imperfect solid lipid core that can accommodate high drug loads. The NLCs exhibit good physical stability, prolonged protection of drug load, improved drug bioavailability [8]. NLCs is also applicable via parenteral route and can maintain prolonged circulating time for as long as 24 h post-intravenous injections [9].

In cancer therapy, targeted drug delivery systems enhance therapeutic efficacy and improved drug sustainability at diseased tissues. Cancer tissue targeting can be achieved with the use of natural or synthetic ligands that interact specifically with receptors on cancer cells. Among receptors that can serve as targets for ligand or drug-loaded carriers are erythropoietin (EpoR), folate, and epidermal growth receptors [10, 11].

Erythropoietin (EPO), produced primarily by the kidneys, is responsible for bone marrow erythrocyte production. EpoRs are not only abundant in erythrocyte precursors but also in cancer cells. Among cancers cells shown to express functional EpoRs include human breast carcinoma (MCF-7), hepatoma (HepG2), osteosarcoma (U2–OS), cervical carcinoma (HeLa), VHL-deficient renal clear cell carcinoma (RCC4), colon carcinoma (HCT-116), and colon carcinoma (7860-WT and SW480) cell lines [12]. However, the status of EpoR on rat mammary gland tumor (LA7) cells is not clear.

Our previous study showed that combination EPO and tamoxifen (TAM) treatments produced greater rat mammary tumor regression than TAM alone [13]. Also, a stable nanostructured lipid carrier system was developed for TAM with improved drug solubility [14]. Based on these observations, we developed the EPO-coated TAM-loaded lipid nanoparticles (EPO-TAMNLC) to enhance the anti-tumor effect of TAM. EPO-TAMNLC showed greater *in vitro* cytotoxicity than TAM on MCF-7 cells [15]. In the current study, the effects of EPO-TAMNLC and TAMNLC on LA7 cells and LA7 cell-induced rat mammary gland tumor were determined. This study also determined the safety of EPO-TAMNLC and TAMNLC as therapeutic compounds using the non-tumorigenic human breast epithelial (MCF-10A) cell line and normal health rats.

## Materials and methods

### Materials

Hydrogenated palm oil (Condea Chemie, Hamburg, Germany) was a gift from the Malaysian Palm Oil Board (MPOB) and olive oil was purchased from Basso Fegele and Figli Srl (San Michele di Serino, Italy). Others ingredients included were polysorbate 80 (Thermo Fisher Scientific, Waltham, MA, USA) Ultrapurified water (Merck Millipore, Billerica, MA, USA) and Lipoid S100 (lecithin) (Lipoid GmbH, Ludwigshafen, Switzerland).

Thimerosal, sorbitol, bovine serum albumin (BSA), dimethyl sulfoxide (DMSO), Tamoxifen free base, 4',6-diamidino-2-phenylindole (DAPI), propidium iodide (PI), ribonuclease A (Rnase A), thiazolyl blue tetrazolium bromide (MTT), Harris’s haematoxylin and eosin (H&E), ketamine hydrochloride, xylazine hydrochloride, horse serum, epidermal growth factor (EGF), hydrocortisone and insulin were purchased from Sigma-Aldrich (St Loius, MO, USA). Recombinant human erythropoietin was purchased from Peprotech (Rocky Hill, NJ, USA), paraformaldehyde (Acros Organics, USA), normal saline (0.9% NaCl), and 10% buffered formalin and Triton X–100 from Thermo Fisher Scientific (United States).

Rat mammary gland tumor cell (LA7) and non-tumorigenic breast (MCF-10A) cells were purchased from the American Type and Culture Collection (ATCC, Manassas, VA, USA).

### Preparation of EPO-coated TAM-loaded nanostructured lipid carrier

As described in our earlier study [12], EPO-TAMNLC was prepared by high-pressure homogenization method. The amount of EPO coat on TAMNLC nanoparticle surfaces was determined via sodium dodecyl sulphate polyacrylamide gel electrophoresis. The thermodynamic interaction between EPO and TAMNLC was characterized using the fluorescent quenching assay and isothermal titration calorimetry (ITC).

### Cell culture

LA7 cells were cultured in phenol-red free RPMI medium (Nacalai Tesque, Japan) supplemented with 10% fetal bovine serum (Thermo Fisher Scientific, United States) and 1% penicillin-streptomycin (Nacalai Tesque, Japan). The MCF-10A cells were cultured in DMEM/F12 (Thermo Fisher Scientific, United States) supplemented with 5% horse serum, 20 ng mL^-1^ epidermal growth factor (EGF), 0.5 mg mL^-1^ hydrocortisone, 10 μg mL^-1^ insulin and 1% penicillin-streptomycin. All cells were cultured in culture flasks with their respective complete medium and incubated in the 5% CO_2_ humidified Binder BD53 incubator (New York, USA).

### Elucidation of estrogen and erythropoietin receptors in LA7 cells

To determine the estrogen receptor-α (ERα) and EpoR status, the LA7 cells were cultured in a 6-well plate (Nunc Nalgene, USA) and harvested upon reaching 70% confluency. The cells were rinsed twice with phosphate buffered saline (PBS) and fixed with 4% (w/v) paraformaldehyde for 20 min. The fixed cells were then rinsed twice with PBS and permeabilized using 0.1% (w/v) Triton–X 100 solution for 10 min. Subsequently, the cells were blocked with 1% (w/v) BSA solution and incubated for 1 h. The cells were then incubated with rabbit anti-ERα (sc542-AF488) (Santa Cruz Biotechnology, USA) and anti-EpoR primary antibodies (sc697) (1:500 in 1% BSA solution) (Santa Cruz Biotechnology, USA) overnight at 4°C. Anti-rabbit antibody (sc3739) (Santa Cruz Biotechnology, USA) tagged with phytoerythrin was added as the secondary antibody and the plate incubated for a further 2 h at room temperature. The cells were rinsed trice with PBS, counter-stained with 4, 6-diamidino-2-phenylindole (DAPI) solution (10.5 μg mL^-1^) for 10 min, and visualized with IX73 Live Cell Imaging fluorescent microscope (Olympus, Japan) with fluorescent excitation of 350, 490 and 550 nm, and emission at 475, 520 and 565 nm, respectively.

### *In vitro* cytotoxic effect of EPO-TAMNLC

#### Cell cytotoxicity assay

LA7 and MCF–10A cells were cultured in T-75 flasks (Nunc Nulgene, USA). Upon reaching 80% confluency, the cells were rinsed with PBS and harvested with 0.05% (w/v) trypsin containing 0.02% (w/v) EDTA. A total of 2 × 10^3^ LA7 and 6 × 10^3^ of MCF-10A cells, each in 100 μL suspension, were seeded into 96-well microplates (TPP, Switzerland). After 24 h incubation, the LA7 cells were treated with 100 μL EPO, EPO + TAM, EPO-TAMNLC, TAMNLC, TAM, and DMSO at five concentrations ranging from 2.5 to 40 μM (2.5 to 40 IU for EPO). In the case of EPO + TAM treatment, EPO was added first to the cells followed by TAM 12 h later. The MCF-10A cells were treated with either 20 μM EPO-TAMNLC, TAMNLC or TAM for 72 h only. For treated LA7 cells, at 0, 24, 48, or 72 h, 20 μL 5 mg mL^-1^ MTT solution were added to each well and the plate incubated for 3 h to allow for the formation of formazan crystals. The supernatant in the wells were carefully removed and the crystals solubilized with 200 μL DMSO. The absorbances were determined at 570 nm and 630 nm using the Biotek EL800 microplate reader (Biotek, USA). The half-growth inhibitory concentration (GI_50_), total growth inhibition concentration (TGI) and lethal concentration 50 (LC_50_) were obtained based on the corrected absorbances (A) calculated using the following formulae:

$$Corrected\;Absorbance\;(A)={Absorbance}_{570\;nm}-{Absorbance}_{630\;nm}$$

$$cell\;viability\;(\%)=\frac{A_{treated}-A_{t0}}{A_{untreated}-A_{t0}}\times 100\%$$

$$cell\;viability\;(\%)=\frac{A_{treated}-A_{t0}}{A_{t0}}\times 100\%$$

Where,

$$A_{treated}\;=absorbance\;of\;treated\;well$$

$$A_{untreated}\;=absorbance\;of\;untreated\;well$$

$$A_{t0}\;=absorbance\;at\;time\;0$$

The viability of cells was determined using the following formula:

$$cell\;viability\;(\%)=\frac{A_{treated}}{A_{untreated}}\times 100\%$$

#### Apoptosis assay

The mode of cell death for treated LA7 cells was determined using Annexin V- fluorescein isothiocynate (FITC)/propidium iodide (PI) double staining assay (Merck Milipore, United States) according to manufacturer’s instruction with slight modification. 2 mL suspension containing 2 × 10^5^ cells were seeded into each well of a 6-well plate (TPP, Switzerland) and incubated overnight in the 5% CO_2_ humidified Binder BD53 incubator (New York, USA) at 37°C. The LA7 cells were treated for 24 or 48 h with either 5, 10 or 20 μM EPO-TAMNLC, TAMNLC or NLC and incubated in the 5% CO_2_ humidified Binder BD53 incubator (New York, USA) at 37°C. All the cells were harvested and centrifuged at 1000 rpm (Eppendorf centrifuge 5810R, Hamburg, Germany) for 5 min to obtain cell pellets. The cell pellets were washed twice with PBS before mixing with 500 μL cold PBS. 10 μL media binding reagent and 1.25 μL Annexin V-FITC was added to the cells and plates incubated for 10 min at 25°C in the dark. Following incubation, the cells were centrifuged at 1000 rpm (Eppendorf centrifuge 5810R, Hamburg, Germany) and resuspended with 500 μL cold 1× binding buffer and 10 μL PI added. The cell suspensions were analyzed immediately with NovoCyte Flow cytometer (ACEA Biosciences, San Diego, USA) and interpreted by Novo Express software version 1.2.4.

#### Cell cycle assay

2 mL of suspension containing 2 × 10^5^ LA7 cells were seeded into each well a 6-well plate and incubated overnight in the 5% CO_2_ humidified Binder BD53 incubator (New York, USA) at 37°C. The LA7 cells were treated with 5, 10 or 20 μM EPO-TAMNLC, TAMNLC or NLC for 24 or 48 h at 37°C. Untreated cells served as controls. All cells were harvested and washed twice with PBS. The cells were then fixed with ice-cold 70% ethanol and stored at -20°C overnight. Prior to analysis, the cells were washed once with PBS then mixed with 425 μL PBS, 50 μL 1 mg mL^-1^ ribonuclease A (Rnase A) and 25 μL PI. All samples were analyzed immediately with NovoCyte Flow cytometer (ACEA Biosciences, San Diego, USA) and interpreted by Novo Express software version 1.2.4.

### *In vivo* toxicity tolerance of EPO–TAMNLC

#### Animals

Virgin female healthy Sprague-Dawley (SD) rats weighing 180 to 200 g were purchased from Takrif Bistari Enterprise, Malaysia. The rats were kept in plastic cages to acclimatize under 12-h light/dark cycle for one week at 20 to 24°C ambient temperature and 40 to 50% relative humidity. The rats were fed commercial feed pellet and water *ad libitum*. Acute toxicity test was conducted for 14 days on rats at animal house of Faculty of veterinary medicine, University Putra Malaysia, according to the OECD 420 guideline with modifications, to determine the effect of EPO-TAMNLC and TAMNLC. All the rats in the study were humanely euthanized by anaesthetized with a mixture of ketamine-HCI (80 mg/kg BW) and xylazine (10 mg/kg BW) [16] prior to cardiac puncture. Approval for the study was obtained from the Institutional Animal Care and Use Committee (IACUC) University Putra Malaysia (UPM/IACUC/AUP-R016/2016).

#### Treatment of acute toxicity

Forty-eight healthy female rats were randomly assigned to 8 treatment groups of 6 rats per group. Group 1 (control): sham-treated with normal saline solution (0.9% NaCl), Group 2: treated with 5 mg kg^-1^ body weight (BW) NLC, Groups 3, 4, and 5: treated with 1.25, 2.5, and 5 mg kg^-1^ BW EPO-TAMNLC, respectively, Groups 6, 7 and 8: treated with 1.25, 2.5, and 5 mg kg^-1^ BW TAMNLC, respectively. All treatments were given once via the tail vein. The doses used for EPO-TAMNLC treatments were decided based on the IV injection volume guideline and formula [17]. At the end of the acute toxicity study, the liver, kidneys, and spleen were collected for histological examination. Whole blood, serum, and bone marrow samples were also taken for toxicity determination.

#### Clinical observation and body weight

Treated rats were examined for any physical and clinical signs of toxicity twice a day. The BW, food consumption, coat appearance, and mortality of rats were recorded for 14 days post-treatment.

#### Hematology

Blood were collected into dipotassium ethylenediaminetetraacetic acid (K2-EDTA) tubes (BD Bioscience, United States) and analyzed using the Scil Vet animal blood counter (HORIBA ABX SAS, France) for hemoglobin (Hb) concentration, erythrocyte (RBC), leukocyte (WBC), and thrombocyte counts, mean corpuscular volume (MCV), and mean corpuscular hemoglobin concentration (MCHC) estimations. The hematocrit (PCV) was determined via the microhematocrit capillary tube method (Vitrex medical, Denmark).

#### Serum biochemistry

Blood samples collected in plain tubes (BD Bioscience, United States) were allowed to clot and serum collected by centrifugation (Hettich zent–EBA 20, Germany) for determination of protein, bilirubin, liver enzymes, and renal function parameter concentrations using diagnostic kits (Roche) and a biochemistry analyzer (Hitachi 902, Japan).

#### Bone marrow analysis

The femur was excised and cut into halves with a sterile scalpel blade. Bone marrow samples from the end of the femur were collected using micropipettes containing 50 μL of 3% EDTA in PBS. Bone marrow smears were made and immediately air-dried [18], stained with Wright stain for 3 min before flooding with buffer at pH 6.8 for 7 min [19]. The stained bone marrow slides were gently washed with tap water, left to dry and mounted with cover slips. The myeloid to erythroid ratio (M:E) were determined microscopically from a total of 500 cells counted [20].

#### Histopathology

At the end of the acute toxicity study, the kidneys, liver, and spleen collected were weighed and immediately fixed with 10% formalin for at least 48 h. All the fixed samples were placed in cassettes, dehydrated overnight in the automated tissue processor (Leica ASP300, Germany), embedded in paraffin wax, trimmed and sectioned with microtome and placed on slides. All the slides were stained with Hematoxylin-Eosin [21]. The sections were viewed under light microscopy.

### Anti-rat mammary gland tumor effect of EPO-TAMNLC

#### Preparation of LA7 mammary gland tumor cells

The LA7 cells were maintained in RPMI-1640 medium supplemented with 10% fetal bovine serum (Gibco, South America), L-glutamine and 1% penicillin-streptomycin, then cultured in a 5% CO_2_ incubator at 37°C. The cells were harvested, counted using a hemocytometer, centrifuged at 1000 rpm (Eppendorf centrifuge 5810R, Hamburg, Germany) for 5 min to obtain cell pellet. The cell pellet was resuspended in PBS and used within 1 h of preparation.

#### Mammary gland tumor induction

Fifty-four healthy female rats were intraperitoneally (IP) anesthetized with a mixture of ketamine-HCI (80 mg/kg BW) and xylazine (10 mg/kg BW) [16]. 300 μL cell suspensions in PBS containing 6 × 10^6^ LA7 cells was administered subcutaneously into the mammary fat pad of each rat using a syringe with 25G needle. Tumor formation were closely monitored for 2 weeks [13]. Treatments with EPO-TAMNLC and TAMNLC at 1.25, 2.5 and 5 mg kg^-1^ were instituted once the tumor sizes reached 0.8 to 0.9 cm diameter [22]. The rats were subjected to euthanasia if achieved weight loss more than 20% start from the beginning and maximum tumor diameter 2 cm. All the rats in the study were humanely euthanized by anaesthetized with a mixture of ketamine-HCI (80 mg/kg BW) and xylazine (10 mg/kg BW) [16] prior to cardiac puncture.

#### Experimental design and drug treatment

Sixty rats were equally divided into one group of healthy non-treated negative control and 9 groups of mammary gland tumor-bearing rats treated with either of the following; 0.3 mL 0.9% NaCl only (tumor control), 2 mg kg^-1^ BW TAM in corn oil, 5 mg kg^-1^ BW NLC, 5 mg kg^-1^ BW, 2.5 mg kg^-1^ BW or 1.25 mg kg^-1^ BW EPO-TAMNLC, 5 mg kg^-1^ BW, 2.5 mg kg^-1^ BW, or 1.25 mg kg^-1^ BW TAMNLC. The EPO-TAMNLC and TAMNLC treatment doses were based on the results obtained in the acute toxicity test. Except for TAM that was given *per os*, all treatments were administered via the tail vein, once per week for 4 weeks. Tumor size and body weight were determined every week post-treatment. The TAM treatment dose was determined based on the following human equivalent dose (HED) formula [23]:

$$HED\;\left(\frac{mg}{kg}\right)=Animal\;dose\;\left(\frac{mg}{kg}\right)\times \frac{Animal\;Km}{Human\;Km}$$

K_m_ factor is defined as body weight (kg) divided by body surface area (BSA) (m^2^) and the value is the average BSA calculations for humans and animals. Using 60 kg as the standard human body weight and the recommended TAM oral dose of 20 mg per day [24], the equivalent animal dose is calculated as follows:

$$(20mg/60kg)=Animal\;dose\;(\frac{mg}{kg})\times (\frac{6}{37})$$

$$Animal\;dose=2\;mg\;{Kg}^{-1}\;BW\;{day}^{-1}$$

#### Tumor mass measurement and histopathology

The mammary gland tumor volume (mm^3^) (length × width^2^) was measured using a digital caliper. Determination of tumor histopathology is as described in the acute toxicity section. Tumor growth activity was determined using the mitotic index analysis (MIA) by randomly counting the number of mitotic events at 400× magnification, where the highest count in 10 fields (HPF) was used as the MIA. The mitotic scores were graded as G1 (0 to 6 mitoses), G2 (7 to 12 mitoses), or G3 (≥13 mitoses) [25].

#### Statistical analysis

All the data obtained were analyzed using one-way of analysis of variance (ANOVA). The analysis was followed by *Post Hoc* multiple comparisons for multiple set of data at α = 0.05.

## Results

### Estrogen and erythropoietin receptors on LA7 cell

**Fig 1:**
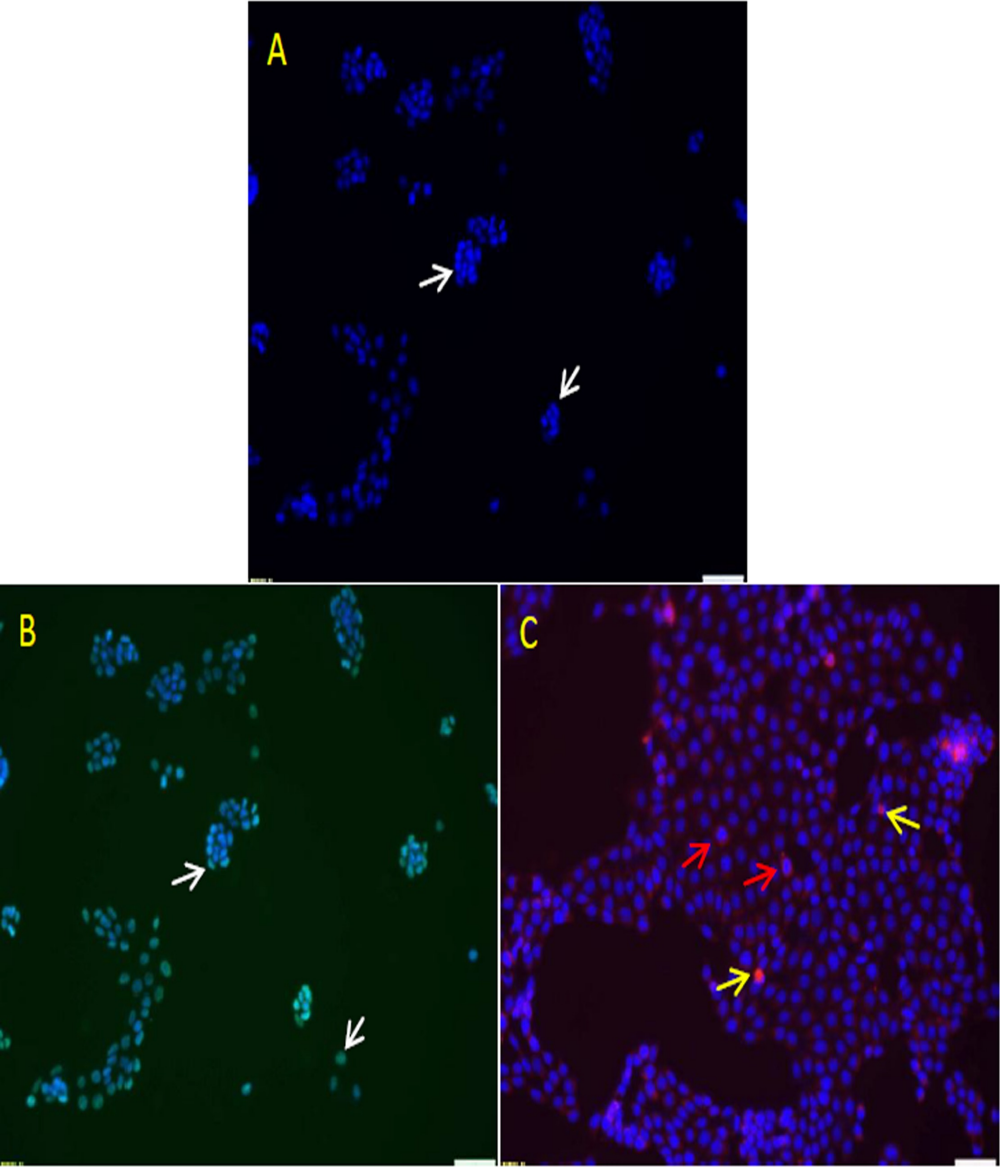
Fluorescence images of estrogen receptor-α (ERα) and erythropoietin receptor (EpoR) in LA7 cells. A—DAPI stain (blue, arrow); B—ERα (green) (arrow); C—EpoR (red, red and yellow arrows). LA7 cells show moderate intensity of ERα expression in the nucleus (white arrow) while EpoR is mainly located around the nucleus (red arrow) and occasionally in the nucleus (yellow arrow). (200×).

LA7 cells were positive for ERα and EpoR. The ERα was located at the nucleus while the EpoRs were in the cytoplasm and around the nucleus (Fig 1).

### In vitro cytotoxic effect of EPO-TAMNLC on LA7 and MCF-10A cells

#### Antiproliferative effect

**Fig 2:**
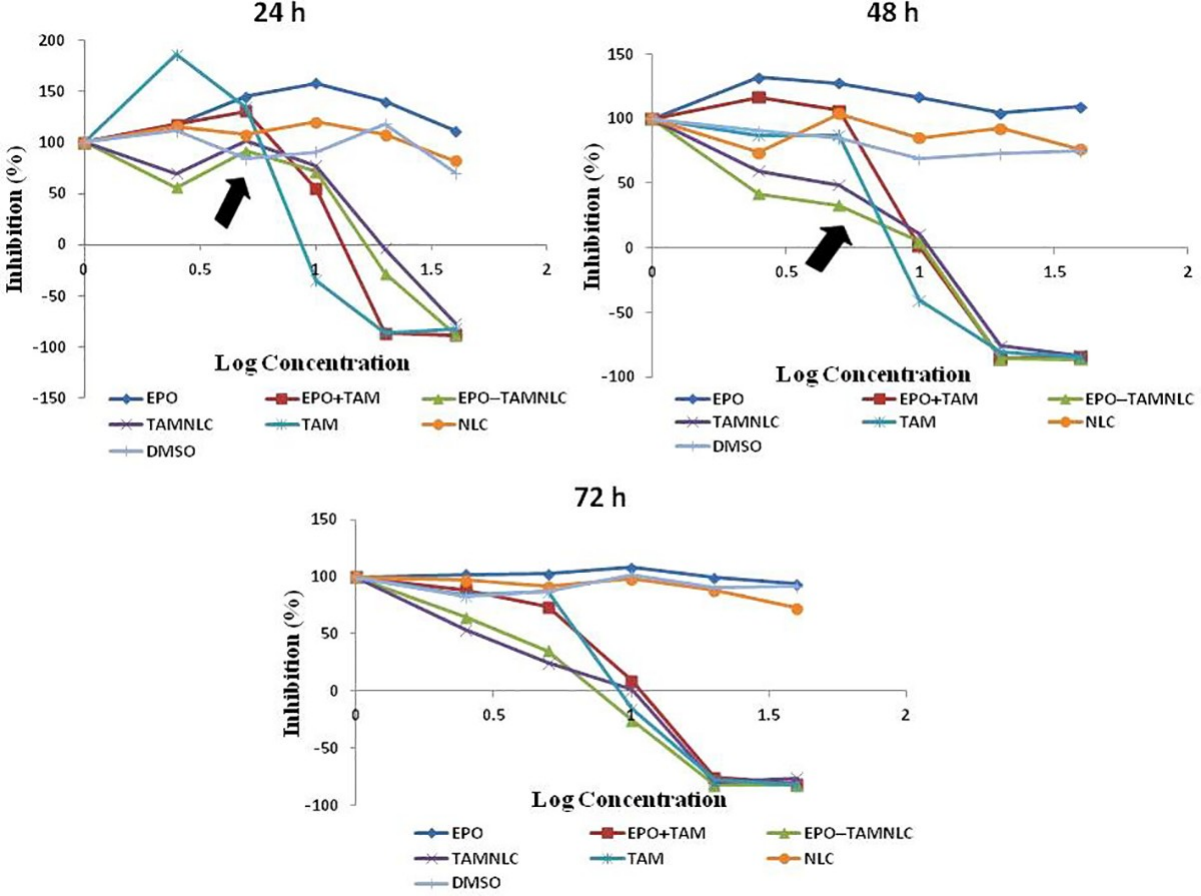
**The concentration-response curve for treated LA7 cells at (A) 24, (B) 48, and (C) 72 h**. The LA7 proliferation inhibition induced by TAMNLC and EPO-TAMNLC was greater 48 and 72 h than 24 h (black arrows). Each point is the average of three independent values. EPO = erythropoietin; TAM = tamoxifen; DMSO = dimethyl sulfoxide. EPO-TAMNLC = tamoxifen-loaded erythropoietin-coated nanostructured lipid carrier; TAMNLC = tamoxifen-loaded nanostructured lipid carrier.

**Fig 3:**
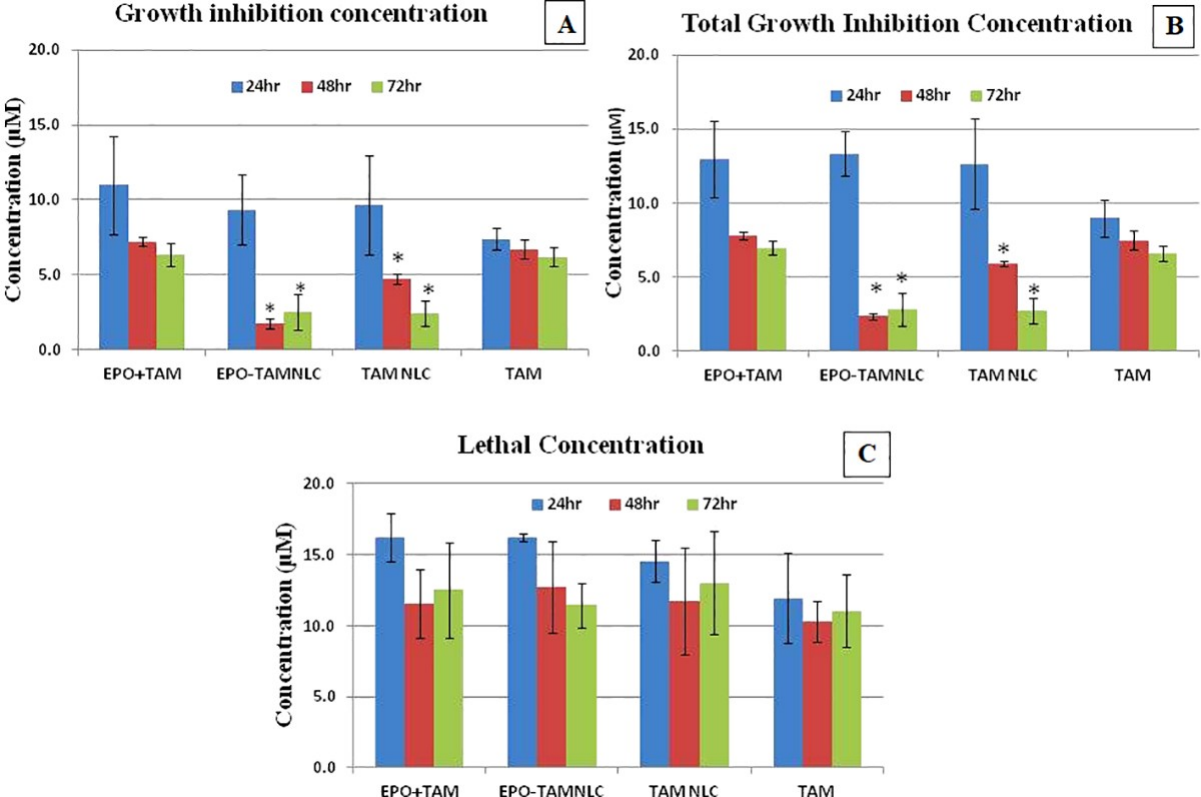
Effect of EPO–TAMNLC on the viability on LA7 as determined by MTT assay with parameters. (A) Growth inhibition concentration, (B) total growth inhibition concentration, and (C) lethal concentration. Each point represents the mean ± std dev. *significantly difference between both EPO+TAM and TAM (p<0.05). TAM = tamoxifen; EPO = erythropoietin; EPO-TAMNLC = tamoxifen-loaded erythropoietin-coated nanostructured lipid carrier; TAMNLC = tamoxifen-loaded nanostructured lipid carrier. EPO-TAMNLC and TAMNLC caused greater reductions in GI_50_ and TGI values at 48 and 72 h than either EPO + TAM or TAM treatments.

EPO-TAMNLC, TAMNLC, TAM, and combination EPO and TAM treatments, all showed dose- and time-dependent cytotoxic effects on LA7 cells (Fig 2). Both EPO-TAMNLC and TAMNLC showed significantly (*p* < 0.05) greater cytotoxic effects on LA7 cells than either EPO + TAM or TAM after 48 and 72 h. EPO-TAMNLC and TAMNLC treatments caused significant greater (*p*<0.05) decreases in growth inhibition concentration (GI_50_) value and total growth inhibition concentration (TGI) after 48 and 72 h in comparison to EPO + TAM and TAM (Fig 3). The results suggest that EPO-TAMNLC and TAMNLC treatment have greater and longer therapeutic effects on the LA7 cells. There was no difference in cytotoxic effect between EPO-TAMNLC and TAMNLC. The lethal concentration (LC_50_) values in LA7 cells did not vary significantly (*p*>0.05) with treatments and time of exposure.

**Fig 4:**
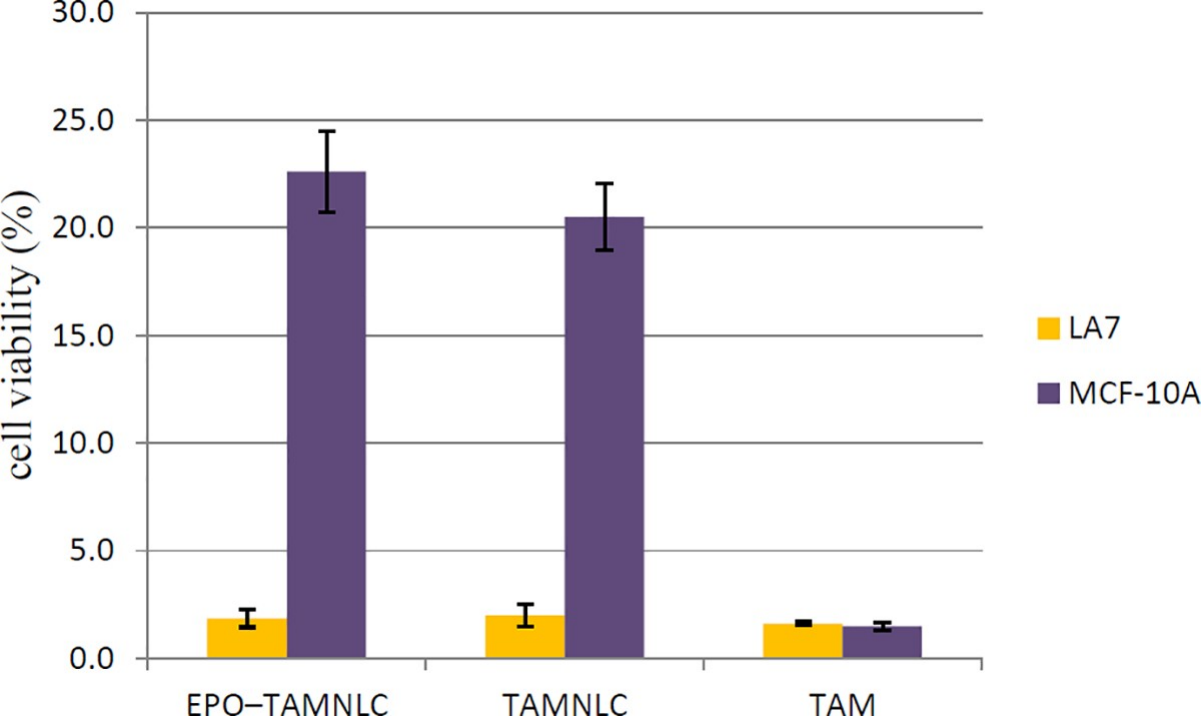
Viability of LA7 and MCF-10A cells treated with 20 μM EPO-TAMNLC, TAMNLC and TAM after 72 h. The viability of treated non-tumorigenic MCF–10A was 10-fold higher than the tumorigenic LA7 cells. Each point represents the mean ± std dev. TAM = tamoxifen, EPO-TAMNLC = tamoxifen-loaded erythropoietin-coated nanostructured lipid carrier; TAMNLC = tamoxifen-loaded nanostructured lipid carrier.

Normal MCF-10A cells were used as controls and to determine the safety and selectivity of treatments. EPO-TAMNLC and TAMNLC was approximately 10 times more cytotoxic to LA7 than MCF-10A (Fig 4). TAM decreased the viability of both the LA7 and MCF-10A cells. There is no significant difference in the effect of TAM between these cell lines. The results suggest that incorporation of TAM into NLC and coating of TAMNLC with EPO significantly increased the cytotoxic effect of TAM towards LA7 cells while remaining relatively innocuous to normal cells. Incorporation into NLC also markedly reduces the toxic effects of TAM on normal cells.

#### Apoptosis assay

**Fig 5:**
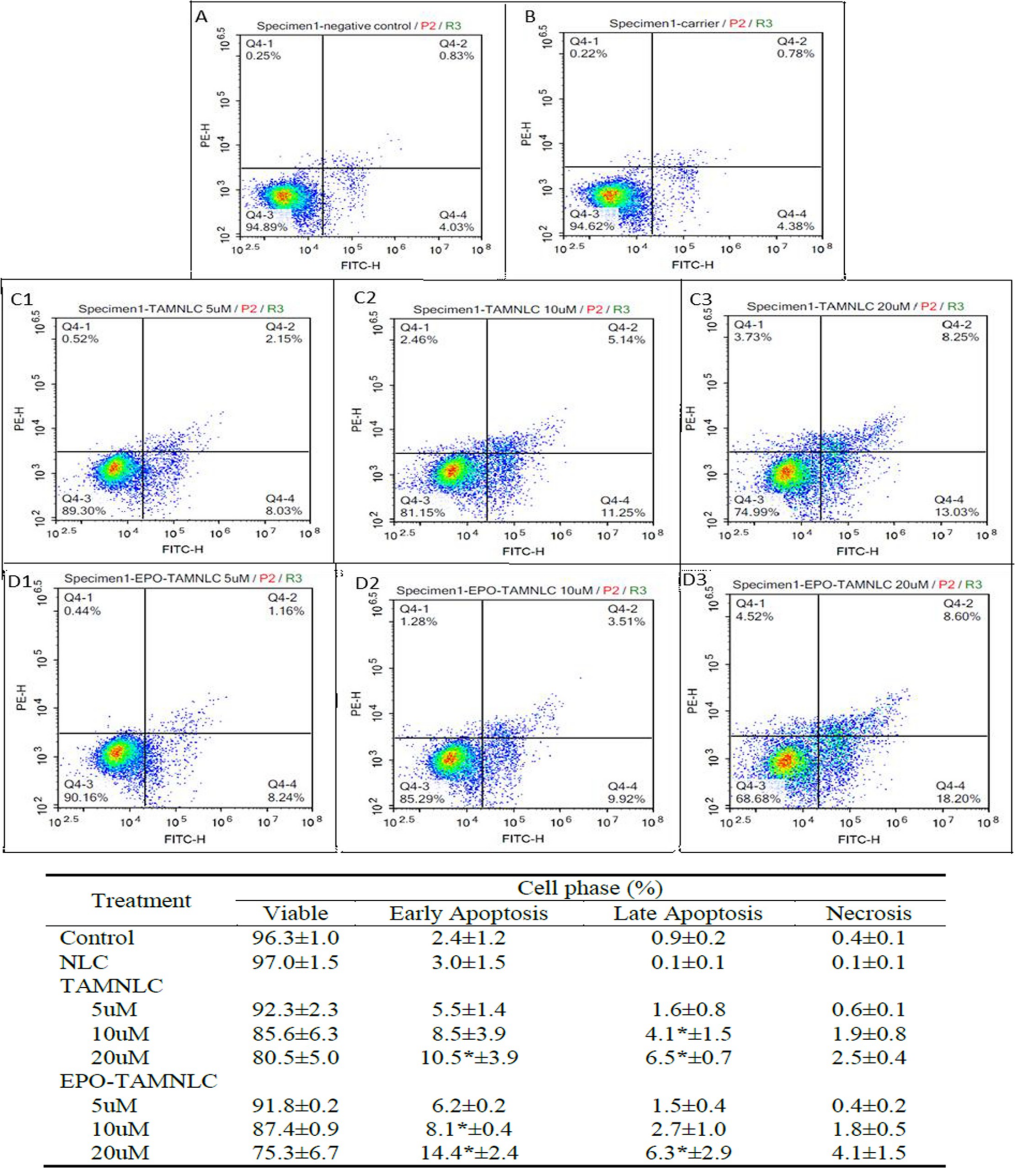
Flow cytometric analysis of LA7 cells treated with EPO-TAMNLC and TAMNLC at 24 h. (A) Negative control, (B) NLC (carrier), (C1) 5, (C2) 10, (C3) 20 μM TAMNLC, and (D1) 5, (D2) 10, (D3) 20 μM EPO-TAMNLC. Cells were stained with FITC–conjugated Annexin V and PI. For each box, lower left quadrant = viable cells; lower right = early apoptotic cells; upper right = late apoptotic cells, and upper left = necrotic cells. The apoptotic effects of EPO-TAMNLC and TAMNLC increased with increase in treatment concentrations. More cells were in early than late apoptosis. Values are mean ± std dev. *For each treatment concentration, significantly difference were in comparison with negative control (p<0.05). NLC = nanostructured lipid carrier; EPO-TAMNLC = tamoxifen-loaded erythropoietin-coated NLC; TAMNLC = tamoxifen-loaded NLC.

**Fig 6:**
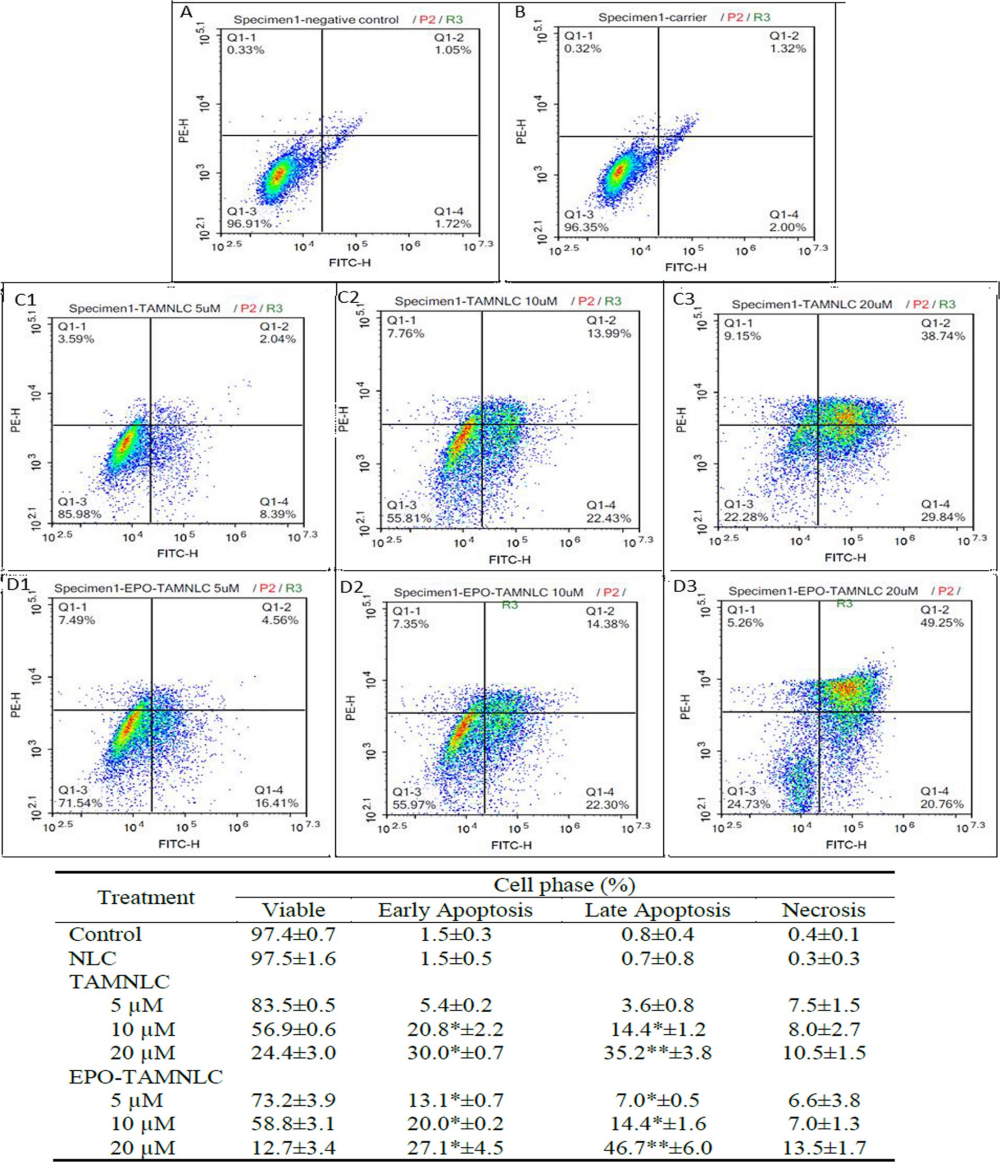
Flow cytometric analysis of LA7 cells treated with EPO-TAMNLC and TAMNLC at 48 h. (A) Negative control, (B) NLC (carrier), (C1) 5, (C2) 10, (C3) 20 μM TAMNLC, and (D1) 5, (D2) 10, (D3) 20 μM EPO-TAMNLC. Cells were stained with FITC-conjugated Annexin V and PI. For each box, lower left quadrant = viable cells; lower right = early apoptotic cells; upper right = late apoptotic cells, and upper left = necrotic cells. The apoptotic effects of EPO-TAMNLC and TAMNLC increased with increase in treatment concentrations. At high treatment concentrations more cells were in late than early apoptosis. Values are mean ± std dev. *For each treatment concentration, significantly differences were in comparison with negative control and NLC treatment (p<0.05). **For each treatment concentration, significantly difference in comparison with EPO–TAMNLC treatment. NLC = nanostructured lipid carrier; EPO-TAMNLC = tamoxifen-loaded erythropoietin-coated NLC; TAMNLC = tamoxifen-loaded NLC.

EPO-TAMNLC and TAMNLC caused significant (p<0.05) time- and dose-dependent apoptosis of LA7 cells, particular at higher doses (Figs 5 and 6). EPO-TAMNLC caused LA7 cell apoptosis earlier and at low concentrations than TAMNLC. By 48 h of 20 μM of EPO-TAMNLC and TAMNLC treatments, more cells were in late than early apoptosis. The study showed that EPO-TAMNLC caused more LA7 cells to enter late apoptosis than TAMNLC.

#### Flow cytometry–based cell cycle assay

**Fig 7:**
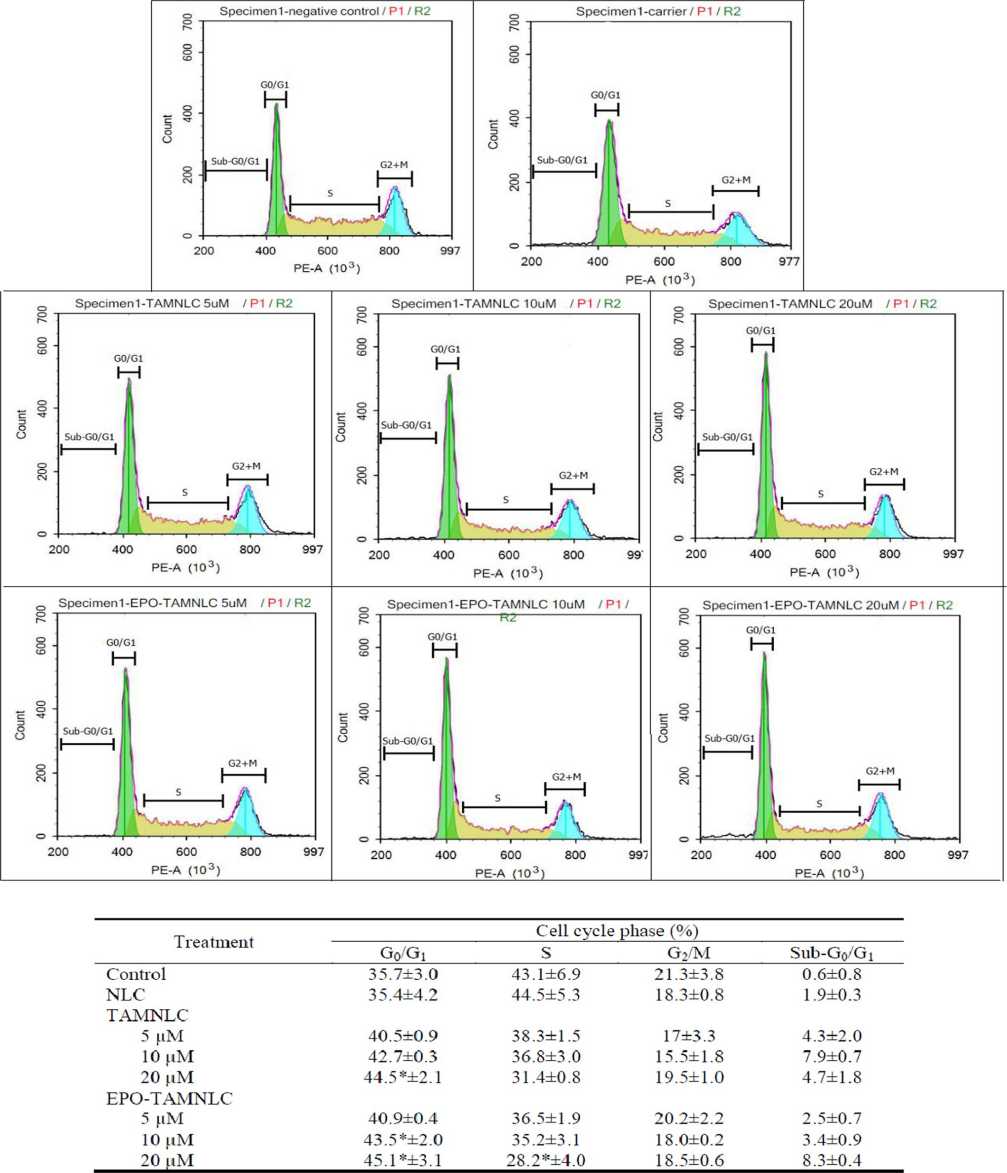
Cell cycle profile of treated LA7 at 24 h. (A) Negative control, (B) NLC (carrier), (C1) 5, (C2) 10, (C3) 20 μM TAMNLC, and (D1) 5, (D2) 10, and (D3) 20 μM EPO-TAMNLC. The population of LA7 cells at G_0_/G_1_ phase increased after EPO-TAMNLC and TAMNLC treatment. Values are mean ± std dev. *For each treatment concentration, significantly differences were in comparison with negative control (p<0.05). NLC = nanostructured lipid carrier; EPO-TAMNLC = tamoxifen-loaded erythropoietin-coated NLC; TAMNLC = tamoxifen-loaded NLC.

**Fig 8:**
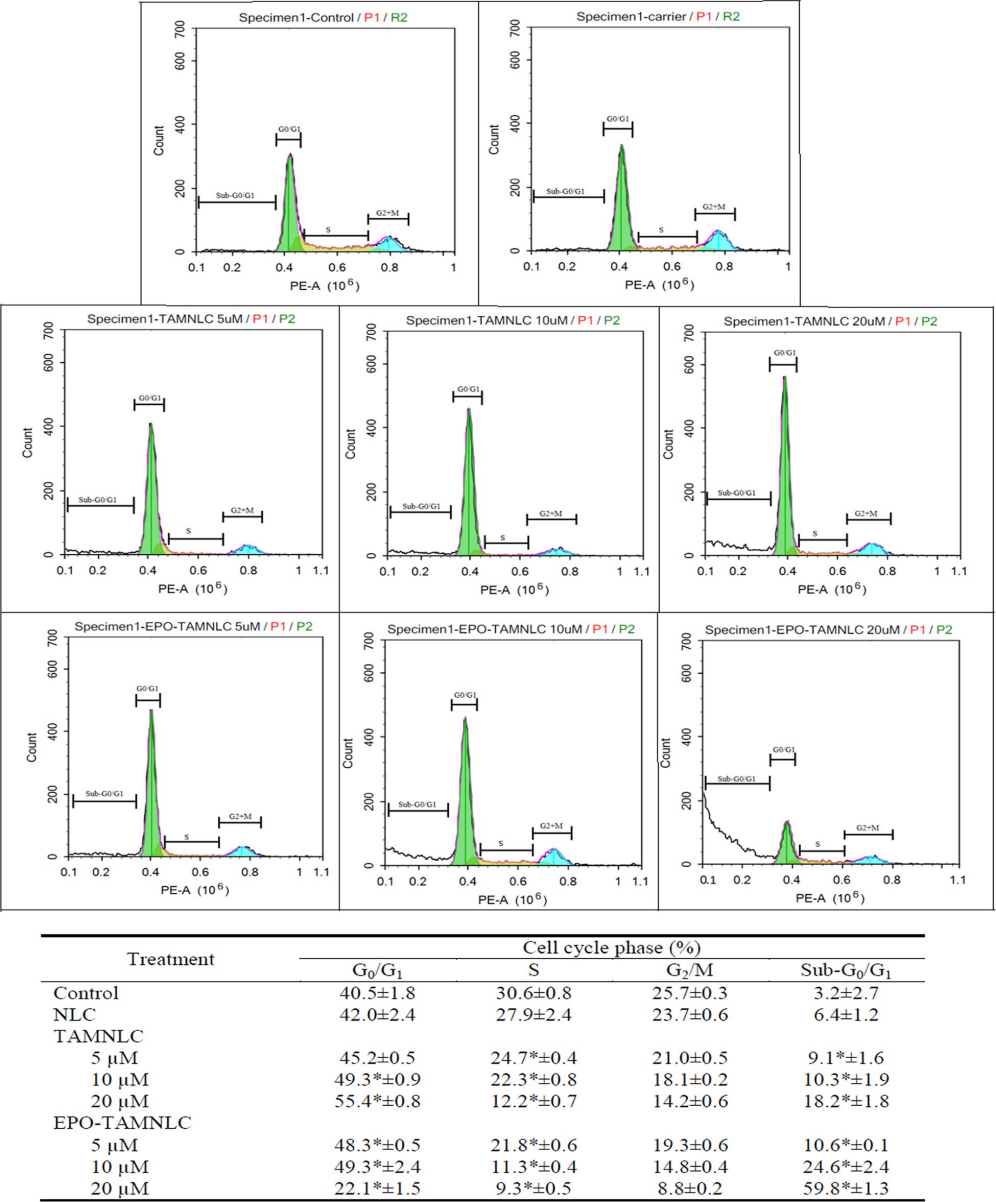
Cell cycle profile of treated LA7 at 48 h. (A) Negative control, (B) NLC (carrier), (C1) 5, (C2) 10, (C3) 20 μM TAMNLC, and (D1) 5, (D2) 10, and (D3) 20 μM EPO-TAMNLC. The LA7 cell population at G_0_/G_1_ and sub-G_0_/G_1_ phase generally increased after EPO-TAMNLC and TAMNLC treatments. However, at 20 μM, EPO-TAMNLC caused a shift of cell population from G_0_/G_1_ to sub-G_0_/G_1_ phase suggesting increase in apoptotic effect. Values are mean ± std dev. *For each treatment concentration, significantly differences were in comparison with negative control (p<0.05). NLC = nanostructured lipid carrier; EPO-TAMNLC = tamoxifen-loaded erythropoietin-coated NLC; TAMNLC = tamoxifen-loaded NLC.

EPO-TAMNLC and TAMNLC treatments caused significant increases (*p*<0.05) in LA7 cell population entering the G_0_/G_1_ phase of the cell cycle (Figs 7 and 8) except for 20 μM EPO-TAMNLC at 48h where a shift of cell population accumulated in sub-G_0_/G_1_ was observed. The effect was more significant (p<0.05) at high treatment doses and generally greater after 48 than 24 h exposure. Treatment of LA7 cells with EPO-TAMNLC and TAMNLC for 48 h caused cells to enter the sub-G_0_/G_1_ or apoptotic phase. The apoptotic effect of EPO-TAMNLC and TAMNLC was concentration dependent, increasing with increase in treatment concentration. EPO-TAMNLC also caused more LA7 cell to undergo apoptosis than TAMNLC.

### Acute toxicity profile of EPO-TAMNLC on Sprague Dawley rat

#### Clinical observation and body weight measurements

**Fig 9:**
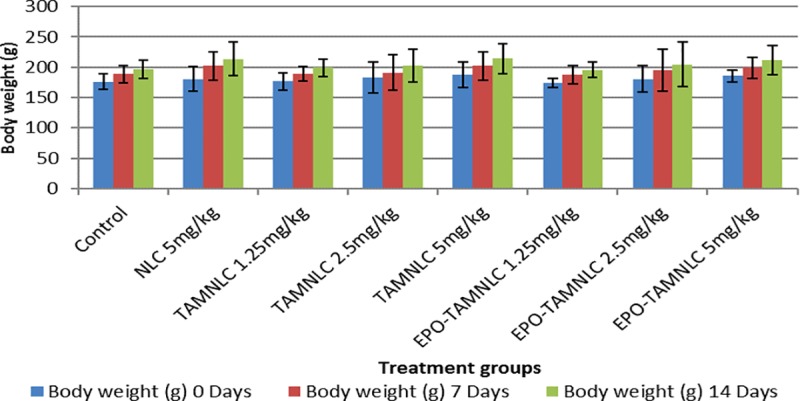
Body weight of rats treated with NLC, TAMNLC and EPO-TAMNLC. No significant difference (*p*>0.05) among groups. Values are mean with std dev. error bars.

The majority of rats did not showed any sign of toxicity, behavioral abnormality or weight loss as the result of treatments. In fact, the body weights in all treated rats increased after 14 days of treatment (Fig 9).

#### Hematological profile

There were no significance difference hematological profiles among groups of rats except for the WBC counts in EPO-TAMNLC treated groups. The WBC counts increased significantly on EPO-TAMNLC 1.25 and 2.5 mg/kg BW treatment groups. EPO-TAMNLC treatment was observed to cause higher total RBC and WBC counts than TAMNLC in rats (Table 1). The effect is suggested to be due to the pro-erythropoietic effect of EPO.

**Table 1:** Hematological parameters in rats treated with tamoxifen–loaded erythropoietin–coated nanostructured lipid carrier, tamoxifen–loaded nanostructured lipid carrier and nanostructured lipid carrier.

Treatment	RBC (x10^12^/L)	Hb (g/L)	PCV (L/L)	MCV (fL)	WBC (x10^9^/L)	Neut (x10^9^/L)	Lymp (x10^9^/L)	Mono (x10^9^/L)	Eosin (x10^9^/L)	Baso (x10^9^/L)	Platelets (x10^9^/L)
Control	7.1±0.4	131±11.6	0.4±0.0	58±2.6	8.60±4.0	2.4±0.8	9.9 ±1.2	0.7±0.3	0.2±0.1	0	657±237
NLC											
5.0 mg kg^-1^ BW	7.2±0.8	136±16.8	0.4±0.1	59±3.2	9.36±3.0	2.2±0.5	9.3 ±2.3	0.5±0	0.2±0.0	0	813 ± 91
TAM–NLC											
1.25 mg kg^-1^ BW	7.5±0.6	141±11.0	0.4±0.0	58±2.6	11.18±5.0	2.0±0.7	10.1±3.3	0.7±0.2	0.2±0.0	0	967± 87
2.5 mg kg^-1^ BW	7.4±0.9	133±19.4	0.4±0.1	53±5.6	11.80±3.0	1.4±0.4	10.4±1.7	0.6±0.3	0.1±0.0	0	902±134
5.0 mg kg^-1^ BW	7.6±0.6	139± 9.0	0.4±0.0	55±3.9	13.30±5.0	1.9±0.9	11.1±3.3	0.7±0.3	0.2±0.0	0	998±134
EPO–TAMNLC											
1.25 mg kg^-1^ BW	8.3±0.5	150± 7.0	0.4±0.0	56±1.7	17.70*±4.0	3.3±1.1	12.9±2.2	0.8±0.4	0.2±0.0	0	977±114
2.5 mg kg^-1^ BW	8.5±0.7	157±14.0	0.5±0.0	57±1.7	19.50*±6.0	2.6±0.8	13.6±3.6	1.0±0.3	0.3±0.0	0	770 ± 95
5.0 mg kg^-1^ BW	8.5±0.6	161±10.2	0.5±0.0	59±3.7	11.61±4.0	2.6±0.8	10.5±2.4	0.6±0.2	0.2±0.1	0	890±102

All data are expressed as mean±SD (n = 6). No significance difference (p>0.05) among parameters. RBC = red blood cell (erythrocyte); Hb = haemoglobin; PCV = packed cell volume (haematocrit); MCV = mean corpuscular volume; WBC = white blood cells (leucocytes); Neut = neutrophil; Lymp = lymphocytes; Mono = monocytes; Eosin = eosinophils; Baso = basophils. TAM = tamoxifen; EPO = erythropoietin; EPO–TAMNLC = tamoxifen–loaded erythropoietin–coated nanostructured lipid carrier; TAMNLC = tamoxifen–loaded nanostructured lipid carrier.

*For each treatment concentration, significantly differences were in comparison with negative control (p<0.05)

#### Serum biochemical profile

EPO-TAMNLC and TAMNLC treatments did not cause any abnormal change in serum biochemical profiles in rats except for urea and AST parameters (Tables 2 and 3). EPO-TAMNLC 2.5 and 5.0 mg/kg BW showed significantly decrease of urea parameters. Also, there were significant decrease of AST parameter in TAMNLC 5 mg/kg BW and EPO-TAMNLC treatment groups respectively.

**Table 2:** Renal function parameters of rats treated with tamoxifen-loaded erythropoietin-coated nanostructured lipid carrier, tamoxifen-loaded nanostructured lipid carrier, and nanostructured lipid carrier.

Treatment	Creatinine (μmol/L)	Urea (mmol/L)	Sodium (mmol/L)	Potassium (mmol/L)	Chloride (mmol/L)
Control	53.0± 3.7	9.3±1.2	153.0±2.0	5.1±0.6	108.0±2.0
NLC 5.0 mg kg^-1^ BW	51.0± 4.4	8.1±0.6	152.0±3.0	4.7±0.3	107.0±4.0
TAMNLC					
1.25 mg kg^-1^ BW	49.0± 3.5	8.5±1.9	152.0±5.0	4.4±0.5	105.0±6.0
2.5 mg kg^-1^ BW	50.0± 2.9	7.6±0.6	148.0±3.0	4.7±0.4	106.0±2.0
5.0 mg kg^-1^ BW	45.0±10.5	6.7±1.0	152.0±1.0	4.5±0.5	107.0±2.0
EPO-TAMNLC					
1.25 mg kg^-1^ BW	51.0±6.7	7.6±1.5	149.0±4.0	4.7±0.7	106.0±3.0
2.5 mg kg^-1^ BW	49.0±3.6	6.6*±0.9	148.0±5.0	4.5±0.7	106.0±4.0
5.0 mg kg^-1^ BW	46.0±5.0	6.1*±0.9	148.0±4.0	4.4±0.3	105.0±2.0

All data are expressed as mean± std dev. No significance difference (p˃0.05) among parameters. NLC = nanostructured lipid carrier; EPO–TAMNLC = tamoxifen-loaded erythropoietin-coated NLC; TAMNLC = tamoxifen-loaded NLC.

*For each treatment concentration, significantly differences were in comparison with negative control (p<0.05)

**Table 3:** Liver function parameters of rats treated with nanostructured lipid carrier, tamoxifen–loaded nanostructured lipid carrier and tamoxifen–loaded erythropoietin–coated nanostructured lipid carrier.

Treatment	Albumin (g/L)	Total Protein (g/L)	Total Bilirubin (umol/L)	Conjugated Bilirubin (umol/L)	ALP (U/L)	ALT (U/L)	AST (U/L)
Control	31.6±2.6	73±4.1	1.4±0.5	0.41±0.2	210±10.7	69±10.5	194.1±17.3
NLC							
5.0 mg kg^-1^ BW	29.1±3.2	68.3±7.9	0.9±0.6	0.3±0	169±25	53±7.2	249±21.2
TAMNLC							
1.25 mg kg^-1^ BW	30.1±3.9	68.3±8.2	0.7±0.4	0.51±0.2	170±31.1	48±11.5	193±26.4
2.5 mg kg^-1^ BW	29.9±2.6	68.7±4.7	0.3±0.3	0.51±0.1	139±5.6	56±19.3	215.2±33.7
5.0 mg kg^-1^ BW	26.4±6.4	62.5±14.3	0.7±0.3	0.49±0.2	169±25.2	45±16.6	156.7*±10
EPO–TAMNLC							
1.25 mg kg^-1^ BW	27.5±1.6	66.1±5.6	1.1±0.6	0.7±0.1	160±25.5	68±20.9	154.1*±21.2
2.5 mg kg^-1^ BW	29±2.4	66.5±6.2	1.3±0.5	0.49±0.2	155±17.1	51±16.2	167.2*±15.8
5.0 mg kg^-1^ BW	29±3.1	67.2±6	0.9±0.6	0.51±0.3	170±24.6	44±10.7	133.4*±22.8

All values are expressed as mean±std dev (n = 6). There is no significance d (p>0.05) among means. ALP = Alkaline phosphatase; ALT = Alanine aminotransferase; AST = Aspartate aminotransferace; NLC = Nanostructured lipid carrier; TAMNLC = Tamoxifen–loaded NLC; EPO–TAMNLC = Tamoxifen–loaded erythropoietin–coated NLC.

*For each treatment concentration, significantly differences were in comparison with negative control (p<0.05)

#### Histopathological observation

**Fig 10:**
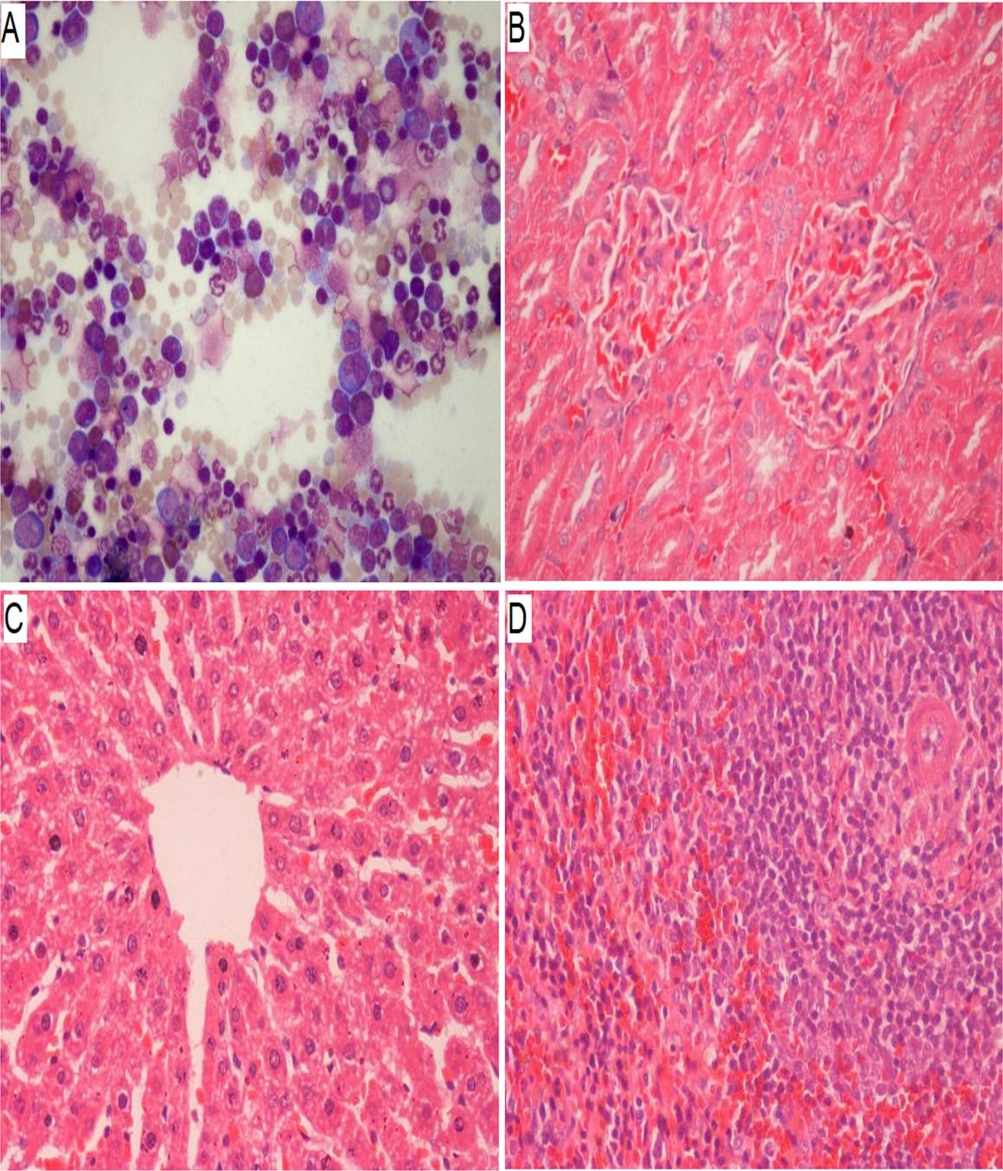
**Representative images of (A) bone marrow, (B) kidney, (C) liver, and (D) spleen tissues of rats treated with EPO-TAMNLC and TAMNLC**. No abnormality was observed in the tissues (400×).

The bone marrow showed normal cellularity, myeloid: erythroid values, cell morphology, and haematopoietic activity (Fig 10). Similarly, no abnormality was observed in the kidneys, liver, or spleen of rats treated with EPO-TAMNLC or TAMNLC.

### In vivo anti–breast effect of EPO–TAMNLC on Sprague Dawley rat

#### Mammary gland tumor induction

**Fig 11:**
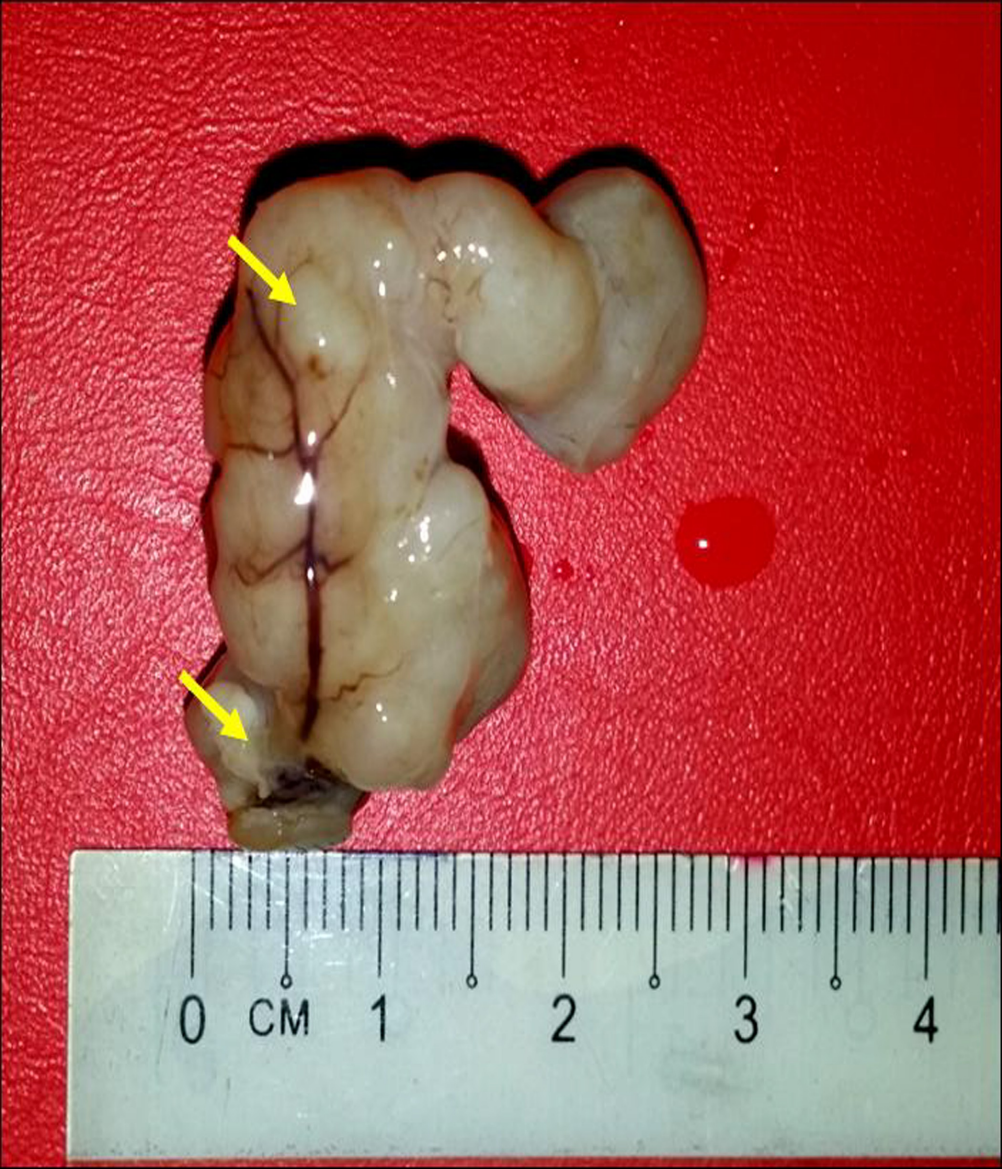
LA7 cell-induced mammary gland tumor in the rat. The tumors are nodular and greyish white in appearance, and well-vascularised which is evident by the thick and engorged blood vessels (yellow arrows).

The mammary gland tumor was successfully induced in the rats. After two weeks the tumors were palpable and of sizes between 500 to 700 mm^3^ (Fig 11). The mammary gland tumor was grossly yellowish white and nodular. Microscopically, the tumor appeared as solid sheets of cells and histologically as poorly differentiated glandular structures and rapidly dividing cells.

#### Antitumor effect of EPO-TAMNLC and TAMNLC

**Fig 12:**
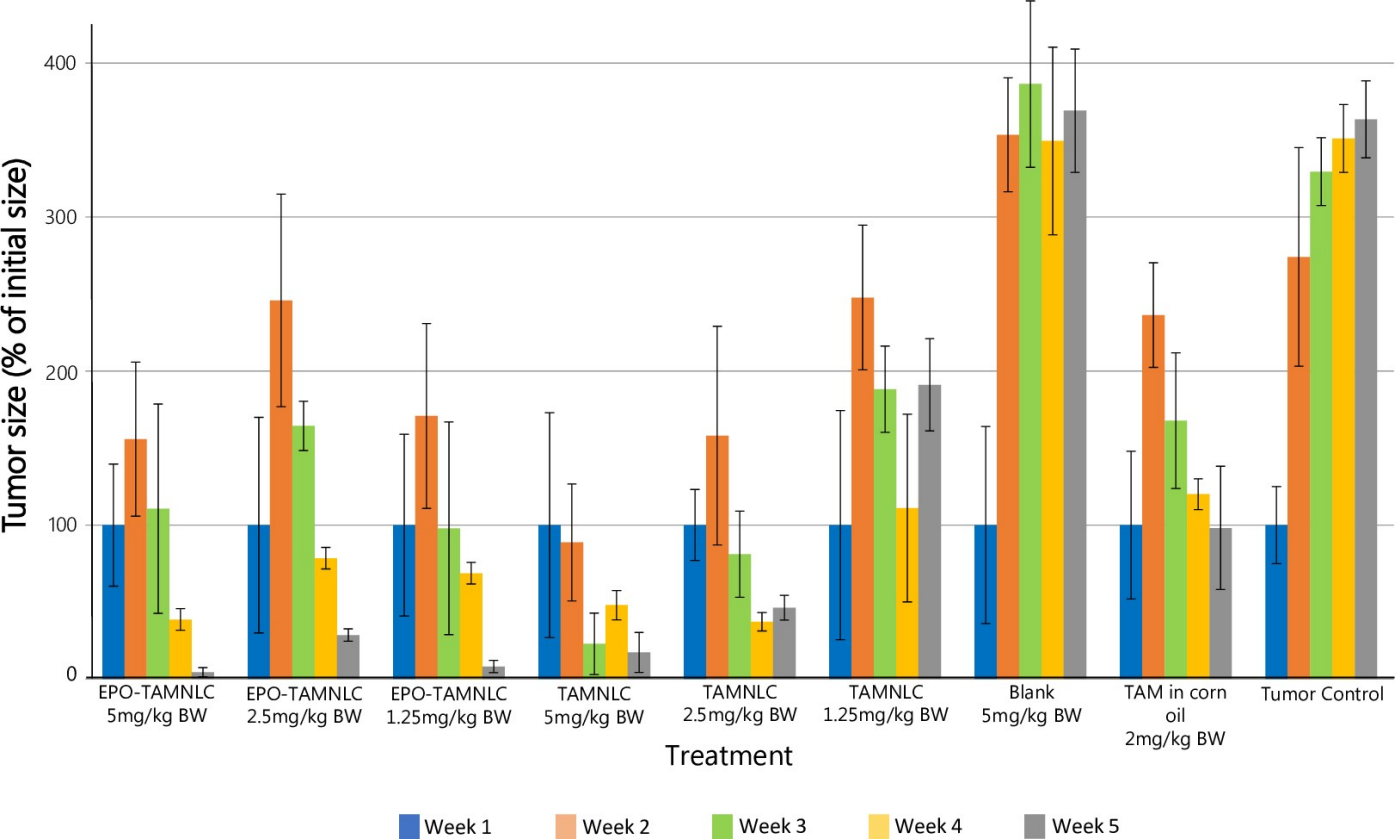
Mammary gland tumor size in rats after treatment with nanostructured lipid carrier, tamoxifen-loaded nanostructured lipid carrier and tamoxifen-loaded erythropoietin-coated nanostructured lipid carrier. EPO-AMNLC and TAMNLC had significantly inhibited growth of rat mammary gland in time–dependent manner. NLC = Nanostructured lipid carrier; EPO–TAMNLC = Tamoxifen–loaded erythropoietin–coated NLC and TAMNLC = Tamoxifen–loaded NLC.

EPO-TAMNLC and TAMNLC showed similar effects in the inhibition of growth of rat mammary gland tumor (Fig 12). All EPO-TAMNLC dosages markedly reduced tumor sizes after two weeks of treatment. The anti-tumor effect of EPO-TAMNLC was sustained, while the effect of TAMNLC was more dose-dependent, with greater effect at higher doses. Tumors from non-treated and NLC-treated rats remained aggressive throughout the study.

#### Histopathology of LA7-induced mammary gland tumor in rats

**Fig 13:**
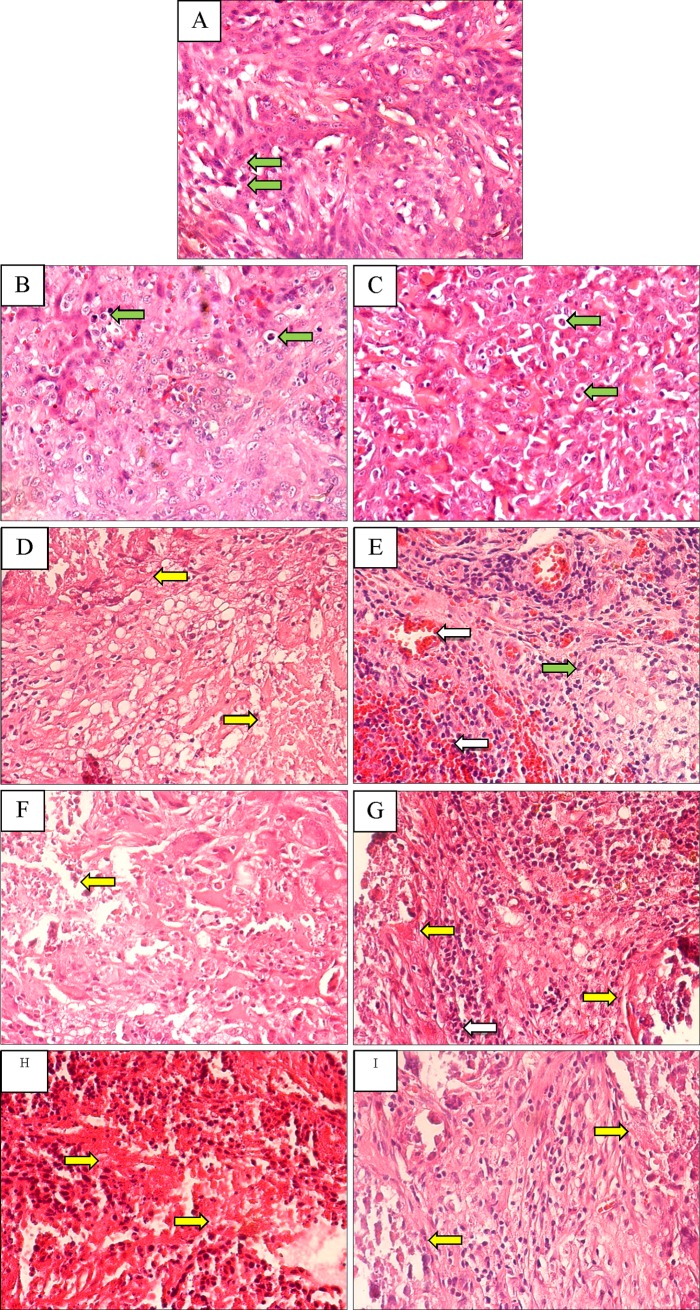
Mammary gland tumor tissue from treated rats. (A) tumor control, (B) intravenous 5.0 mg kg^-1^ BW NLC, (C) oral 2.0 mg/kg BW TAM in corn oil, and intravenous (D) 1.25, (E) 2.5 and (F) 5.0 mg kg^-1^ BW EPO-TAMNLC and (G) 1.25, (H) 2.5, and (I) 5.0 mg kg^-1^ BW TAMNLC The tissues show hyperchromatic tumor cells with abnormal mitotic figures (brown arrows), tumor degenerative area with necrotic tissues (yellow arrows), marked infiltration of mononuclear inflammatory cells, particularly lymphocytes, together with severe vascular congestion (white arrows). NLC = nanostructured lipid carrier; EPO-TAMNLC = Tamoxifen-loaded erythropoietin-coated nanostructured lipid carrier; TAMNLC = Tamoxifen-loaded nanostructured lipid carrier.

The mammary gland tumor sections from rats treated with EPO-TAMNLC were generally characterized by massive degenerative and with necrotic tissues and markedly reduced number of tumor cells (Fig 13). The tumor tissue from the NLC-treated rats consisted of poorly differentiated cells with score 1 nuclear pleomorphism and hyperchromatic tumor cells. The tumor tissues from the control, NLC-, TAM- and TAMNLC-treated rats showed G1 grade mitotic score. No mitotic figure was observed in the mammary gland tumor tissues of rats treated with EPO-TAMNLC and TAMNLC.

## Discussion

NLC-based drug carrier formulations are versatile because they can be administered by various routes, including orally, intradermally, and intravenously. This nanoparticulated carrier system is characterized by high drug payload, good physical stability, high water-solubility [26], prolonged therapeutic effects, and tissue targeting [27]. Loading in NLC increases the solubility and facilitating parenteral application and controlled release of TAM [28].

EPO-TAMNLC was developed to further improve the therapeutic effect of TAM through EpoR targeting [28]. Structurally, EPO-TAMNLC comprises of loaded TAM within the core and a coating of EPO on the surface of the nanoparticles. Since EpoR are highly expressed in breast cancers [12, 29], EPO coating on EPO-TAMNLC surfaces serve as the ligand in the binding of the nanoparticles to EpoR on the breast cancer cells [15]. EPO does not compromise the efficaciousness of breast cancer chemotherapeutics [30, 31]. In fact, in an early study, we showed that EPO-TAMNLC and TAMNLC had greater antiproliferative effect on MCF-7 cells than TAM [15].

EPO-TAMNLC is safe for use in the treatment of breast cancers. This is evident by the *in vitro* studies that showed EPO-TAMNLC is 10 times less toxic to MCF-10A than LA7 cells, while TAM is similarly toxic to these cell lines. Thus, loading of TAM into the EPO-TAMNLC complex did not only increase solubility of TAM but also significantly reduced its toxic effect. Based on GI_50_ and TGI values at 48 and 72 h of exposure, EPO-TAMNLC also showed prolonged and greater anti-LA7 cell effects.

Both EPO-TAMNLC and TAMNLC are toxic to LA7 cells. However, EPO-TAMNLC produced quicker and greater antiproliferative and apoptotic effects on LA7 cells than TAMNLC. The antiproliferative effect of EPO-TAMNLC seemed to also occur via G_0_/G_1_ cell cycle arrest and associated with concurrent reduction of cells in the S phase. The G_0_/G_1_ arrest was similarly to that seen in MCF-7 treated with TAM [32].

EPO-TAMNLC and TAMNLC are safe for parenteral application. The LD_50_ values of EPO-TAMNLC and TAMNLC in rats were greater than 5 mg kg^-1^ BW dosage. We also determined potential toxic effects by estimating blood parameters and tissue changes in rats treated with these drug carrier complexes. None of the rats showed any toxic effect from the treatments. However, rats treated with EPO-TAMNLC showed slightly higher RBC counts that those treated with TAMNLC. This presumably is due to the stimulatory effect of EPO on erythropoiesis. Besides, there were significantly concomitant increases in leucocytes count as the results of EPO-TAMNLC treatment. It seem that the formulation have stimulatory of bone marrow production of blood cells as well. For the renal function profiles, although urea decrease significantly in EPO-TAMNLC treated group, it is still fall within the normal range [33]. With regard to liver function profile, a significant decrease of AST parameter was noted. The increase in the level of AST indicated as increase in liver damage [34]. A decrease in the AST enzymes activity is not considered to give any toxicological significance [35]. Histologically, EPO-TAMNLC and TAMNLC did not cause any abnormal morphological change to the bone marrow, kidney, liver, or spleen tissues, further showing that they are safe for therapeutic use.

The xenograft method is a reliable and fast method for the development rat mammary gland model for the determination of anti-breast cancer effects of candidate therapeutics [36]. In this rat model, intravenously administrated EPO-TAMNLC and TAMNLC produced sustained antitumor effects that were significantly more effective than oral TAM. It is prerequisite that drug candidates are subject of preclinical and clinical trials before they can be marketed for medical use. The rat is one of the commonly used animal models in preclinical studies. The LA7 cell-induced rat mammary gland model is an excellent model for human breast cancer studies. We characterized the LA7 cells to ascertain the suitability of LA7 cell-induced rat mammary gland tumor as a model for determination of the anti-breast cancer properties of EPO-TAMNLC. Immunocytochemistry staining revealed LA7 cells express ERα and EpoR.

Based on the results, it is postulated that EPO-TAMNLC exerts active targeting effect on breast cancers through the EpoRs on breast cancer cells. It is also postulated that uptake of EPO-TAMNLC by cancer cells is through EpoR-mediated endocytosis, otherwise known as clathrin-mediated endocytosis [37]. Although receptor-mediated endocytosis is a saturable process by virtue of the limited number of receptors on cell surfaces, EpoRs in breast cancer tissues are actually much higher in number than in normal breast tissue [38]. Since the EpoR complex lacks intrinsic enzymatic activity, the internalisation of EPO-TAMNLC is proposed to be dependent of JAK2 kinase activity and EpoR cytoplasmic tyrosine [39, 40].

In this study, the usage of EPO was limited due to its tremendously high price. Some studies including *in vitro* cellular uptake through flow cytometry and subchronic toxicity study of EPO-TAMNLC cannot be done. The quantitative *in vitro* study on cellular uptake of EPO-TAMNLC and the further safety evaluation of EPO-TAMNLC in preclinical trials are yet to be investigated in the future.

## Conclusion

The EPO-TAMNLC is a physiochemically stable and efficacious drug delivery system for the treatment of breast cancers. The formulation is safe for parenteral use and does not produce adverse side effect. This delivery system is not only unique because the dual drug loading features, it also has potential for high specificity in target of tissues, especially those positive for ERs and EpoRs. The anti-cancer mechanism of action of EPO-TAMNLC is via the reduction cancer cell proliferation, inducing *in vitro* apoptosis and cancer cell cycle arrest at the G_0_/G_1_ phase.

The effect of intravenous EPO-TAMNLC is more potent than TAM administered orally in *in vivo* studies. In breast cancer therapy, EPO-TAMNLC is expected to effectively inhibit tumour development and progression at doses lower than that required with oral TAM therapy. Loading of TAM in the carrier complex protects normal and non-targeted tissues from the deleterious effects of TAM. This will subsequently reduce the potential of developing side effects while avoiding the development of resistance to the drug.

## Supporting information

S1 File NC3Rs ARRIVE guidelines checklist. Checklist of animal research: reporting of in vivo experiments. PONE-D-18-32607.
(PDF)
